# Early changes in the metabolic profile of activated CD8^+^ T cells

**DOI:** 10.1186/s12860-016-0104-x

**Published:** 2016-07-07

**Authors:** Clemens Cammann, Alexander Rath, Udo Reichl, Holger Lingel, Monika Brunner-Weinzierl, Luca Simeoni, Burkhart Schraven, Jonathan A. Lindquist

**Affiliations:** Institute of Molecular and Clinical Immunology, Otto-von-Guericke-University, Magdeburg, Germany; Max-Planck-Institute for Dynamics of Complex Technical Systems, Magdeburg, Germany; Department of Experimental Pediatrics, Otto-von-Guericke-University, Magdeburg, Germany; Department of Immune Control, Helmholtz Centre for Infection Research, Braunschweig, Germany; Department of Nephrology and Hypertension, Diabetes and Endocrinology, Otto-von-Guericke University, Magdeburg, Germany

**Keywords:** T-cell activation, Aerobic glycolysis, AKT/PKB, Lactate

## Abstract

**Background:**

Antigenic stimulation of the T cell receptor (TCR) initiates a change from a resting state into an activated one, which ultimately results in proliferation and the acquisition of effector functions. To accomplish this task, T cells require dramatic changes in metabolism. Therefore, we investigated changes of metabolic intermediates indicating for crucial metabolic pathways reflecting the status of T cells. Moreover we analyzed possible regulatory molecules required for the initiation of the metabolic changes.

**Results:**

We found that proliferation inducing conditions result in an increase in key glycolytic metabolites, whereas the citric acid cycle remains unaffected. The upregulation of glycolysis led to a strong lactate production, which depends upon AKT/PKB, but not mTOR. The observed upregulation of lactate dehydrogenase results in increased lactate production, which we found to be dependent on IL-2 and to be required for proliferation. Additionally we observed upregulation of Glucose-transporter 1 (GLUT1) and glucose uptake upon stimulation, which were surprisingly not influenced by AKT inhibition.

**Conclusions:**

Our findings suggest that AKT plays a central role in upregulating glycolysis via induction of lactate dehydrogenase expression, but has no impact on glucose uptake of T cells. Furthermore, under apoptosis inducing conditions, T cells are not able to upregulate glycolysis and induce lactate production. In addition maintaining high glycolytic rates strongly depends on IL-2 production.

**Electronic supplementary material:**

The online version of this article (doi:10.1186/s12860-016-0104-x) contains supplementary material, which is available to authorized users.

## Background

T cells play a central role in the immune system and are crucial for the adoptive immune response. Activation of T cells by specific antigens leads to proliferation, differentiation into effector cells, and cytokine production.

A variety of stimuli, including soluble or immobilized antibodies (Abs) that recognize the T cell receptor (TCR), peptide-loaded APCs, or MHC-I tetramers carrying high- or low-affinity peptides, have been used to study T cell responses. It was previously shown that different stimuli lead to either proliferation or apoptosis of thymocytes [1] and mature T cells [2]. However, it is poorly understood how triggering of the same receptor with ligands of different affinity can induce these different outcomes. Since it is known that thymocytes which cannot fulfill their energy demands undergo apoptosis [3] we hypothesized that changes in the metabolic profiles in activated T cells might contribute to cell fate specification.

Stimulation of T cells leads to a change from a quiescent resting state into an activated state, which is characterized by an extensive cell growth, proliferation, and the production of effector proteins, such as cytokines. In the resting state, T lymphocytes maintain their basal energy demands primarily through a mixed usage of glucose and glutamine [3]. However, to meet the increased energy demands following activation, glucose metabolism increases as a source of energy and providing precursor molecules for cellular biosynthesis [4]. Unlike hepatocytes and myocytes, lymphocytes do not have large internal glycogen stores. This makes them highly dependent on extracellular glucose. Glucose uptake in T cells is mediated by the glucose-transporter 1 (GLUT1). It was previously shown that upregulation of GLUT1 expression depends on co-stimulation via CD28 [5, 6]. Co-stimulation is also responsible for the activation of PI3K/AKT, which is thought to be involved in the expression of GLUT1 at the cell surface [7]. However it was shown recently that AKT does not appear to be required for the upregulation of GLUTI and for the increase in glucose uptake upon T cell stimulation [8].

Another important regulator of cellular metabolism is the adenosine-monophosphate kinase (AMPK), which promotes ATP conservation and production through the upregulation of glycolysis, fatty acid oxidation, and the inhibition of ATP-consuming pathways such as protein synthesis, fatty acid synthesis, gluconeogenesis, and glycogen synthesis. AMPK can be activated by an increase in the AMP:ATP ratio followed by phosphorylation through LKB1, a serine/threonine kinase [9–11]. In addition it is known that triggering of the TCR activates AMPK in an AMP-independent, but Ca^2+^-calmodulin-dependent kinase kinase 2 (CAMKK2)-dependent manner, which was shown to activate AMPK independent of AMP levels [12, 13].

We demonstrate here that stimulation of murine CD8^+^ T cells with MHC-I tetramers carrying the high affinity OVA-peptide SIINFEKL leads to the transient activation of AMPK followed by an increase in the glycolytic rate and production of lactate, to counter the increased demand for ATP after activation. Furthermore, we show that the inhibition of lactate production leads to a decreased proliferation. Additionally we confirmed that AKT is required for the glycolytic change in CD8+ T cells whereas mTOR is dispensable. Investigation of later time points revealed a connection between CTLA4 upregulation and downregulation of IL2 production accompanied by subsequent downregulation of lactate production.

## Results

### Antibody stimulation induces ATP depletion, whereas tetramers do not

In our experimental system we activated OT-I T cells using either cross-linked soluble CD3 and CD8 monoclonal antibodies (mAbs) (antibody stimulation) or cross-linked H-2K^b^ molecules loaded with the SIINFEKL peptide (tetramer stimulation). As described in our previous study [2], antibody stimulation leads to a strong and transient activation of signaling molecules downstream of the TCR and induces apoptosis. In contrast, tetramer stimulation shows a weak, but sustained activation of signaling molecules that induces T cell proliferation.

**Fig. 1:**
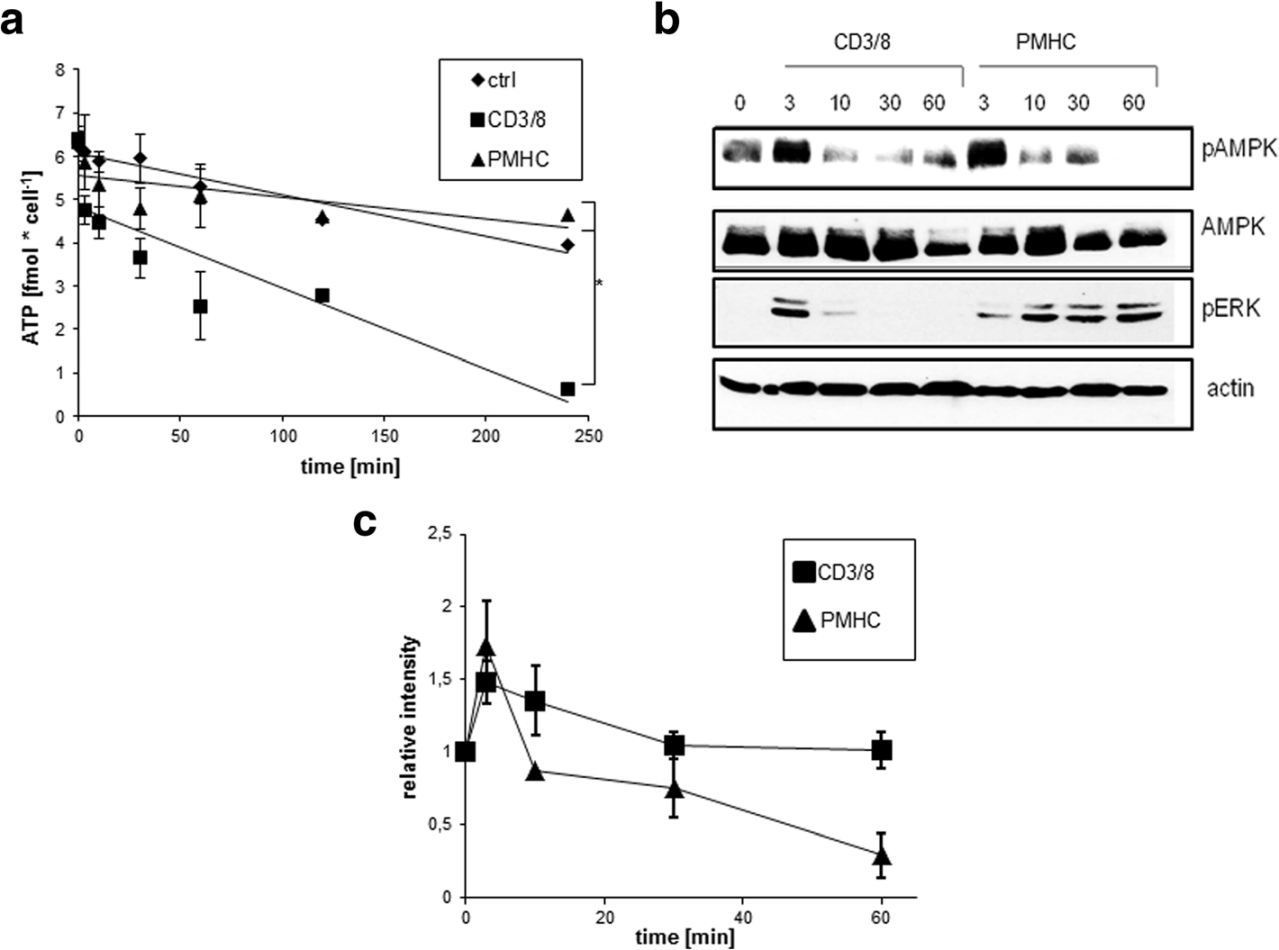
Stimulation of CD8^+^ T cells with antibodies leads to rapid ATP consumption. Purified CD8^+^ T cells were treated with either soluble CD3/CD8 mAbs or OT-I tetramers (PMHC) for the indicated time periods. **a** Cellular ATP production was analyzed using the ATP-Assay Kit. *n* = 6 (**b**) AMPK activation was determined by Western blotting. Phospho-ERK staining was included to show effective stimulation. Total AMPK and actin are included as loading controls. **c** Quantification of AMPK phosphorylation within 60 min after stimulation, *n* = 3

We first assessed whether the concentration of ATP changes upon T cell stimulation by measuring the level of intracellular ATP. Figure 1a shows that within 4 h after stimulation with soluble antibodies, there is a noticeable decrease in the ATP concentration, which is not seen upon tetramer stimulation. Moreover, we confirmed this result by analyzing various metabolites including ATP by mass spectrometry (MS) coupled to high-performance anion-exchange chromatography (data not shown). The observed decrease in the unstimulated control is due to the fact that purified mouse T cells die when left in culture without a stimulus.

We next analyzed the activation of the metabolic regulator AMPK (Fig. 1b), which becomes phosphorylated by LKB1 if the AMP:ATP ratio is increased. Here we show that AMPK is activated immediately by both stimuli. At later time points, antibody-stimulated T cells continue to show a sustained activation of AMPK, whereas tetramer-stimulated cells do not. This led us to conclude that the higher ATP levels observed after tetramer stimulation, result in AMPK inactivation. In contrast, lower ATP levels observed upon antibody stimulation correlate with sustained AMPK activation.

### Tetramer stimulation actives glycolysis, leading to lactate production

**Fig. 2:**
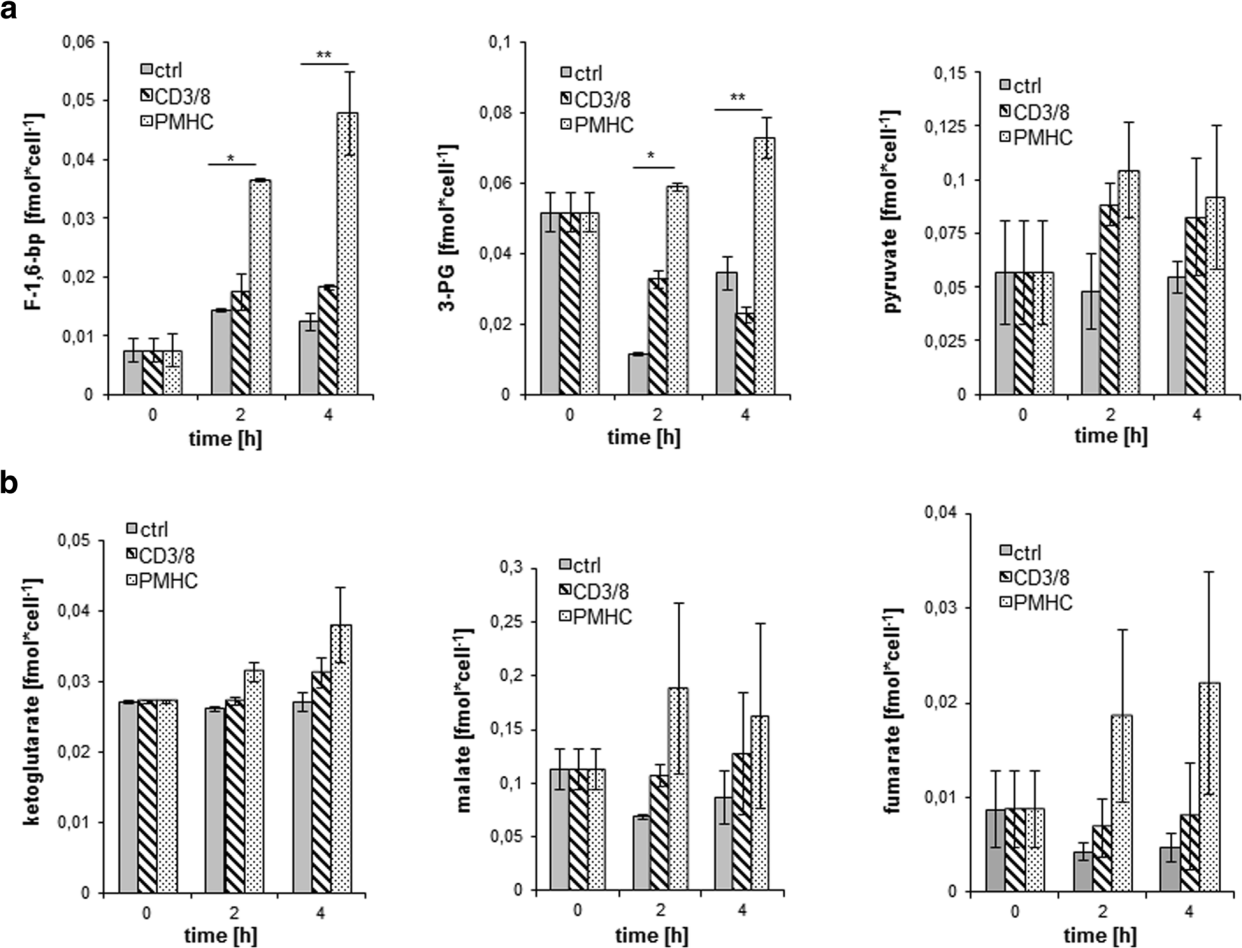
Stimulation of CD8^+^ T cells with tetramers leads to enhanced glycolysis with no significant change in the citric acid cycle. Purified CD8^+^ T cells were treated with soluble CD3/CD8 mAbs or OT-I tetramers for the indicated time periods. Samples were analyzed by MS coupled high-performance anion-exchange chromatography for intermediates of glycolysis – Fructose-1,6-bisphosphate (F-1,6-bp), 3-phosphoglycerate (3-PG) and pyruvate (**a**) and the citric acid cycle – a-ketoglutarate, malate and fumarate (**b**). *n* = 5

It has been previously shown that stimulation of primary human T cells results in the upregulation of glucose uptake and glycolysis [14]. To analyze whether this holds true in our system, we investigated the generation of metabolites for glycolysis and the citric acid cycle. We found that 2-4 h after tetramer stimulation, the concentrations of the glycolytic metabolites fructose-1,6-bisphosphate and 3-phosphoglycerate were significantly increased (Fig. 2a). The concentrations of the end product pyruvate were also slightly, but not significantly increased. For metabolites of the citric acid cycle, we observed a trend towards increased ketoglutarate, malate, and fumarate upon tetramer stimulation (Fig. 2b). In contrast to tetramer stimulation, changes in glycolysis and citric acid cycle were not observed after antibody stimulation.

**Fig. 3:**
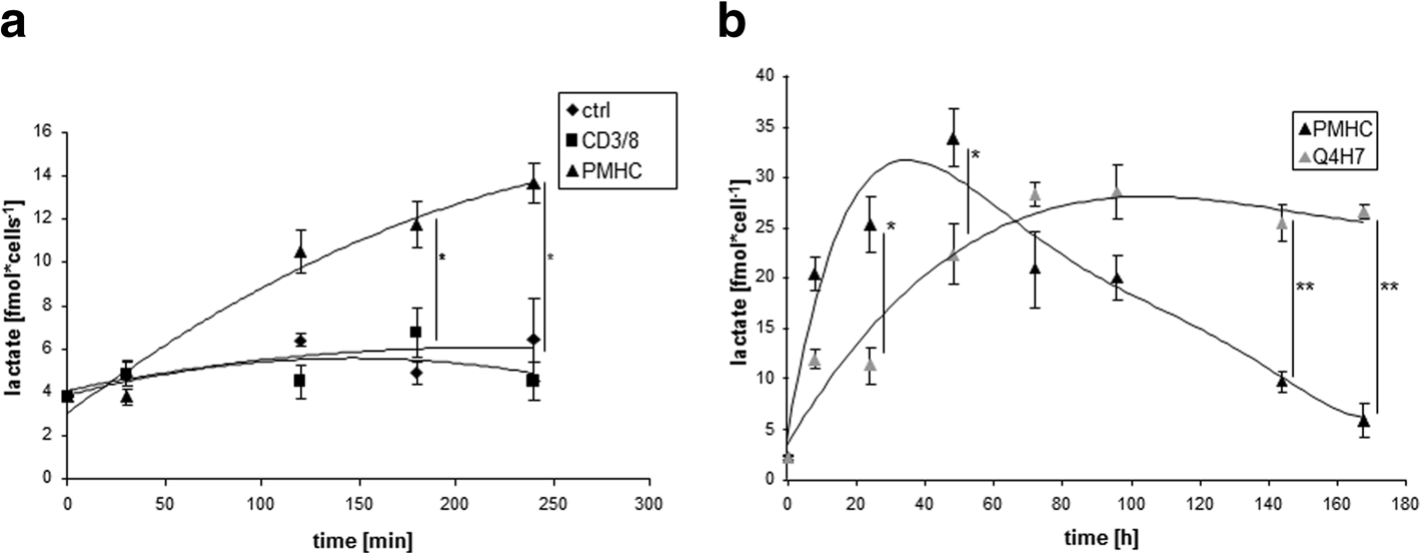
Enhanced glycolysis after stimulation of CD8^+^ Tcells leads to production of lactate. Purified CD8^+^ T cells were stimulated with OT-I tetramers or soluble CD3/CD8 mAbs (**a**) or tetramers carrying the Q4H7 peptide (**b**) for the indicated time periods. Samples were analyzed for production of lactate. *n* = 4

Since we did not observe changes in metabolism upon antibody stimulation, we focused our investigation on the analysis of metabolic parameters upon tetramer stimulation. The observed increase in glycolysis without significant changes in the citric acid cycle lead us to hypothesize that pyruvate produced by glycolysis is converted into lactate (i.e. aerobic glycolysis). To test this hypothesis, we directly measured lactate production (Fig. 3a). We observed a high lactate production after stimulation with tetramers, whereas there was no lactate production after antibody stimulation. When we analyzed later timepoints, we found that lactate production occurs at a high rate during the first 48 h following stimulation. At later timepoints we observed a decrease in lactate production. Since T cells die within 24 h upon antibody stimulation, we used the low affinity peptide Q4H7 as a “negative” control, as this induces survival, but not proliferation [1].

### Proliferation of activated T cells requires both lactate production and functional electron transport chains

**Fig. 4:**
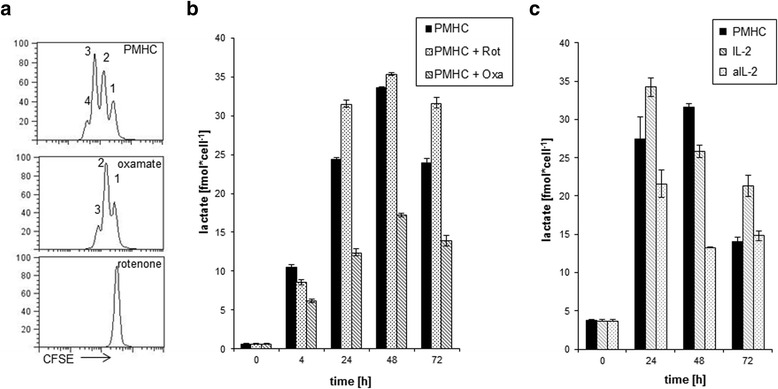
Inhibition of lactate production leads to decreased proliferation/IL-2 is necessary for lactate production. Purified CD8^+^ T cells were treated with OT-I tetramers for the indicated time periods. Samples were analyzed for proliferation (**a**) and lactate production (**b**) in presence of rotenone or oxamate. Samples were analyzed for lactate production in presence of IL-2 or anti-IL-2 (**c**). *n* = 3

To determine if lactate production is required to maintain T cell function we added oxamate, which inhibits lactate dehydrogenase and hence effectively blocks lactate production. We found a decreased proliferation upon the addition of oxamate, thus indicating the necessity of lactate production for T cell proliferation (Fig. 4a). Although we observed no significant changes in the citric acid cycle, the addition of rotenone, which blocks ATP production by interfering with complex I of the electron transport chain in mitochondria, also caused a complete abrogation of proliferation (Fig. 4a). To control the function of the inhibitors rotenone and oxamate we analyzed lactate production. In the presence of oxamate, as expected, we observed a decrease in lactate production, whereas the addition of rotenone showed no effect on lactate produced upon stimulation (Fig. 4b). This led us to the conclusion that even if T cells shift their metabolism towards glycolysis upon stimulation, the TCA cycle is still required. To assess the toxicity of rotenone and oxamate we analyzed the cells for apoptosis (Additional file 1: Figure S1D), where no toxic effects could be observed.

**Fig. 5:**
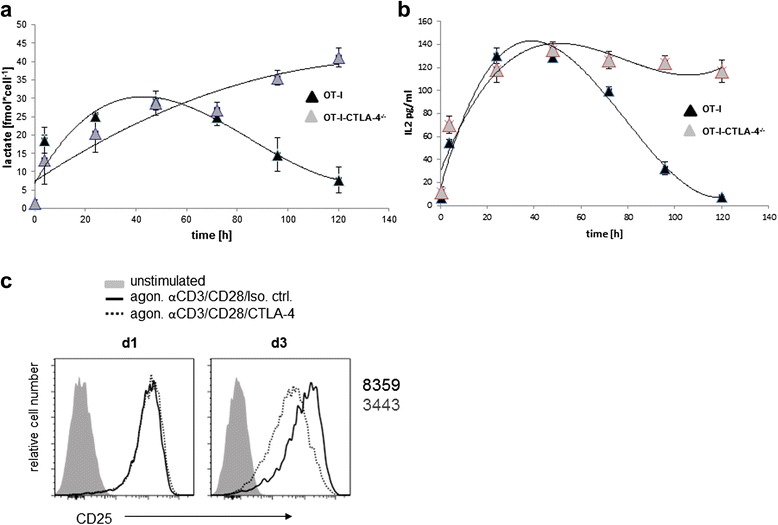
CTLA4-/- OT-I T cells showed no decrease in lactate production and IL-2 production and CTLA-4 mediated reduction of surface CD25 expression in WT CD8 T cells. Purified CD8+ T cells from CTLA-/- or CTL4+/+ mice were stimulated with OT-I tetramers for indicated time points, cells were analyzed for intracellular lactate concentrations (**a**), intracellular IL-2 concentrations by ELISA (**b**), and (**c**) Naïve (CD62Lhigh) CD8+ T cells from WT mice were stimulated with anti-CD3/CD28/CTLA-4 or anti-CD3/CD28/Isotype control-coupled microspheres. Surface expression of CD25 was detected by flow cytometry. Geomean numbers of CD25 fluorescence are depicted for the respective conditions

Since lactate production rapidly decreases 48 h after stimulation, we hypothesized that there must be a switch that shuts off lactate. IL-2 is an autocrine growth factor which drives proliferation at later stages of T cell activation. We tested the hypothesis that IL-2 is involved in switching off lactate production. Therefore we applied a neutralizing IL-2 antibody to our cultures in order to prevent the binding of IL-2 to its receptor. To confirm that our neutralizing IL-2 antibody was effective and not toxic, we analyzed Stat5 phosphorylation 24 h after stimulation (Additional file 1: Figure S1C) and apoptosis (Additional file 1: Figure S1D). Surprisingly, adding of the neutralizing IL-2 antibody to the culture leads to decreased lactate production (Fig. 4c). Moreover the addition of exogenous IL-2 to the stimulated cells fostered the production of lactate (Fig. 4c). This led us to the conclusion that IL-2 is needed to maintain lactate production and does not act as inhibitor of lactate production. We next assessed lactate production in OT1-T cells from CTLA4^-/-^ mice upon stimulation. Interestingly the decrease in lactate production could not be observed in these cells (Fig. 5a). Moreover the loss of CTLA4 leads to a sustained production of IL-2 (Fig. 5b). Additionally, this could be confirmed by adding exogenous CTL4 to the cells, which had an impact on IL-2 receptor expression (Fig. 5c). These results indicate that CTLA4 plays a critical role in modulating IL-2 and lactate production.

### AKT regulates lactate dehydrogenase expression

**Fig. 6:**
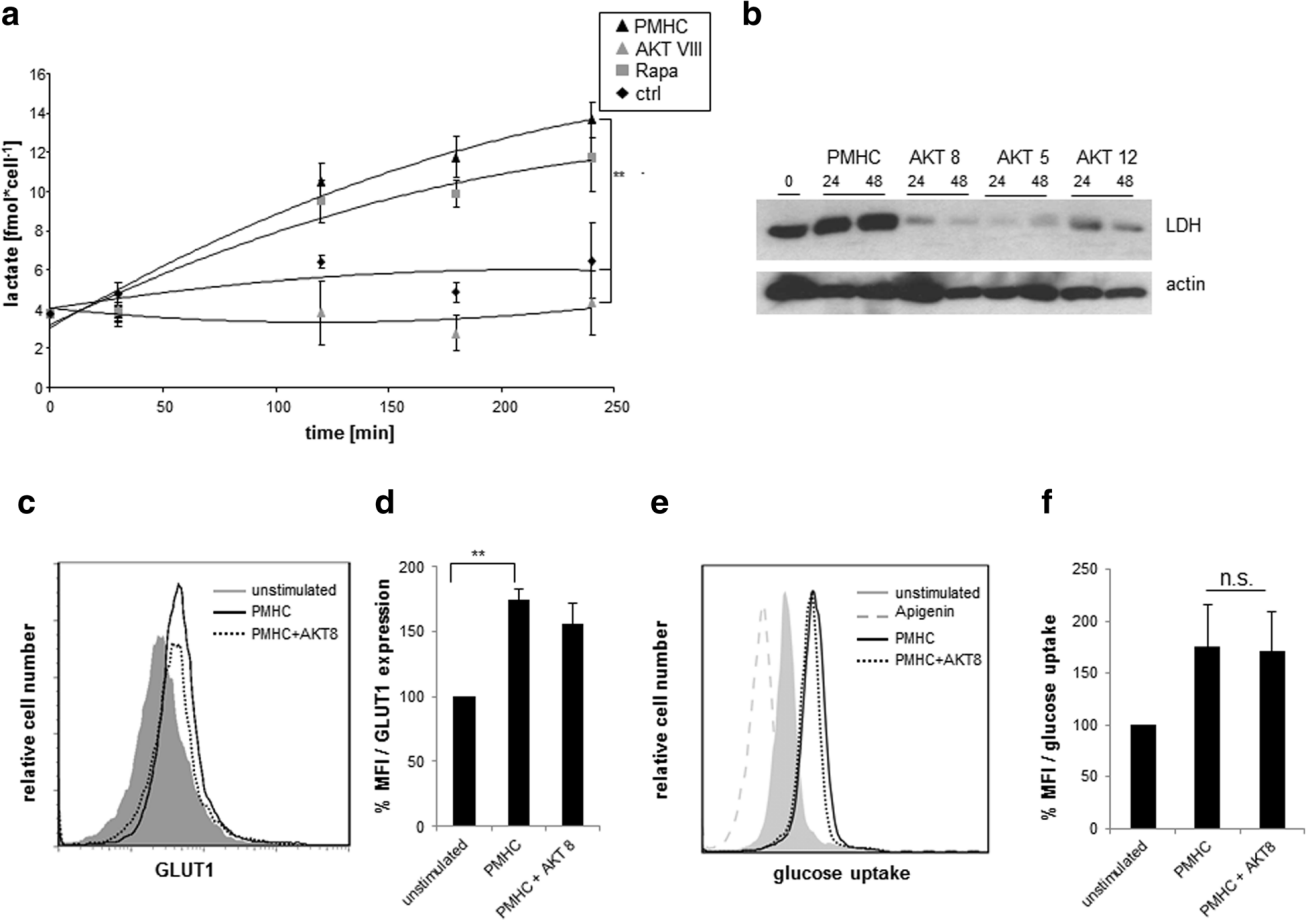
Inhibition of AKT abrogates lactate production. Purified CD8^+^ T cells were treated with OT-I tetramers for the indicated time periods. Samples were analyzed for production of lactate in presence of the AKT-Inhibitor AKT 8 and the mTOR-inhibitor Rapamycin (**a**). Samples were analyzed for upregulation of LDH by Western blotting in the presence of 3 different AKT inhibitors (**b**). Expression of GLUT1 after tetramer stimulation in presence and absence of AKT inhibitor was analyzed via FACS (**c**) and calculated from 3 independent experiments (**d**). Glucose uptake was analyzed 24 h after stimulation of CD8^+^ T cells with tetramers in the absence and presence of AKT inhibitor and apigenine (**e**) and calculated from 4 independent experiments (**f**)

To further analyze whether, in addition to AMPK, other signaling molecules take part in the regulation of metabolism, we investigate AKT and mTOR, which are known to be involved in nutrient sensing in T cells [15]. We applied inhibitors of these enzymes to analyze their relative contribution to lactate production. We observed that both inhibitors block the phosphorylation of p70S6K, which is a downstream target of both mTOR and AKT (Additional file 1: Figure S1A and B) thus confirming that both inhibitors work in our system. We found that the inhibition of the mTOR-complex with rapamycin had no effect on lactate production, whereas the inhibition of AKT leads to a complete abrogation of lactate production (Fig. 6a).

To investigate if the inhibition of lactate production upon AKT inhibition is a direct effect, we analyzed the lactate dehydrogenase (LDH), the enzyme responsible for the conversion of pyruvate to lactate. We observed an increase in total LDH levels after tetramer stimulation which was strongly reduced in the presence of the AKT inhibitor (Fig. 6b).

Another proposed target of AKT in T cell metabolism is GLUT1, which is upregulated upon stimulation to ensure increased glucose uptake. We observe an upregulation of GLUT1 after tetramer stimulation (Fig. 6c and d) However, when we applied the AKT inhibitor we saw no effect on GLUT1 upregulation. We next analyzed the glucose uptake after tetramer stimulation (Fig. 6e). Here, we selectively analyzed the glucose uptake in living cells by including an Annexin V/PI staining to exclude dead and apoptotic cells which do not actively uptake glucose. We confirmed that, upon tetramer stimulation, glucose uptake is increased, but the addition of the AKT inhibitor showed no effect. Hence, we conclude that AKT is not required for the increase of glucose uptake in CD8^+^ T cells. As a negative control we added apigenin which prevents the uptake of the fluorescent glucose.

## Discussion

It was previously described for OT-I mouse T cells that stimulation with soluble antibodies results in apoptosis whereas more physiological stimuli like the stimulation with OT-I tetramers lead to proliferation and differentiation of T cells reflecting the in vivo situation. The reason why these different stimuli have different outcomes on the T cell fate remains still unclear. Therefore we hypothesized that the depletion of the intracellular ATP stores following antibody stimulation could be responsible for the induction of apoptosis. It was previously shown for tumor cell lines that if intracellular ATP concentrations fall below a certain limit, apoptosis is induced [16, 17]. Furthermore several studies showed that apoptotic processes require ATP to transfer apoptotic signals into the nucleus as well as for chromatin condensation and nuclear fragmentation [18–20]. In this study we observed a rapid consumption of ATP upon antibody stimulation, whereas ATP levels remain constant after tetramer stimulation. The question is whether the drop in ATP levels are the cause or a consequence of the resulting apoptosis. First consider that the changes in ATP levels were observed already within the first hour upon stimulation. From this one may assume that the dramatic phosphorylation which occurs upon antibody stimulation leads to a high consumption of ATP [21]. This in turn results in the induction of apoptosis, which further reinforces the reduction in ATP levels. Therefore our results suggest that following tetramer stimulation, T cells switch on metabolic programs to generate ATP in order to counterbalance their ATP consumption.

We next investigated the activation of AMPK, as this is the major energy sensor in cells and its activation is closely linked to intracellular ATP levels. We observed an initial activation of AMPK upon both tetramer and antibody stimulation. This initial activation was previously reported to be induced by the activation of CAMKK2 upon TCR triggering [13]. Since AMPK is further activated by a high AMP:ATP ratio, the decreased ATP levels observed upon antibody stimulation forces AMPK to remain active. In contrast, upon tetramer stimulation, T cells maintain high levels of ATP, thus inactivating AMPK. This led us to the conclusion that tetramer stimulation induces additional changes in the metabolic profiles, since the initial activation of AMPK is not sufficient to shift T cell metabolism towards glycolysis, which is needed to maintain proliferation.

Several previous studies [5, 8, 14] analysed the upregulation of glycolysis by monitoring glucose consumption from the media, direct glucose uptake by the cell, or the upregulation of GLUT1. Here we used a new method to analyze 25 metabolites and nucleotides of glycolysis and citric acid cycle to further investigate the shift towards glycolysis [22]. In our experiments with tetramer stimulation, we clearly observed a significant increase in glycolytic metabolites compared to either the antibody stimulated or unstimulated cells. We also observed small changes in the metabolites of the citric acid cycle, however these were not statistically significant. This led us to the conclusion that aerobic glycolysis is the major energy-producing process after activation.

These observations were confirmed by the fact that, after tetramer stimulation, T cells produce high amounts of lactate, which is an indication for increased aerobic glycolysis, also known as the “Warburg effect”. The fact that lactate production is essential for T cell proliferation was confirmed by the observation that addition of oxamate, a lactate dehydrogenase inhibitor, lead to decreased proliferation. Nevertheless, T cells still require the citric acid cycle to maintain proliferation, as shown by inhibiting the ATP production with rotenone. This inhibition is rather indirect because rotenone blocks complex I of the electron transport chain, disrupting the proton gradient at the mitochondrial membrane, which in the end abolishes the ATP synthase reaction. In a recent study comparing metablic features of T cell subsets, the authors observed striking differences between CD8+ T cells, which showed more glycolytic metabolism, compare to CD4+ T cells, which show higher rates of mitochondrial oxidative metabolism and a greater maximal respiratory capacity [23]. However activation and proliferation of both cell types were similar sensitive to the addition of rotenone. This supports the idea that the TCA cycle is not only required for the generation of ATP, but is also required to deliver substrates for biosynthetic processes like the generation of nucleotides. Furthermore it has been shown that during oxidative phosphorylation, reactive oxygen species are generated which played a critical role in T cell activation [24].

Surprisingly we observed that after 48 h of activation, lactate production decreases. This observation led us to hypothesize that there is a switch in T cell metabolism to shut down aerobic glycolysis. Our hypothesis was that with the transition from antigen-driven proliferation to cytokine-driven proliferation, IL-2 might also be responsible for switching off lactate production and directing pyruvate into the TCA cycle to generate precursors for biosynthesis. When we tested this hypothesis we found that IL-2 is required to maintain lactate production and appears to play no role in switching off lactate. This observation contradicts a previous study were the authors showed that the removal of IL-2 within the first 20 h of stimulation had no effect on lactate production in CD4+ primary human T cells [5]. On the other hand it is known that IL-2 can induce negative regulators of T cell activation like CTLA4, which downmodulates T cell responses in order to prevent an overreaction of the immune system [5, 25]. Interestingly, when we assessed lactate production in OT1-T cells from CTLA4^-/-^ mice, the decrease in lactate production could not be observed indicating that CTLA4 plays a critical role in modulating the switch of glycolytic end products. Furthermore this could be correlated to the IL-2 production in these cells. Under normal conditions the expression of IL-2 ultimately leads to the upregulation of CTLA4 [26, 27]. This in turn leads to feedback inhibition of IL-2 production, which would explain the observed decrease in lactate production [28]. Furthermore the addition of exogenous CTLA4 to the cells leads to reduced CD25 expression, which confirms the role of CTLA4 in regulating IL-2. The possible corresponding ligand triggering surface CTLA4 in CD8+ T cells was shown to be CD80, which is also present on activated T cells serving as a T cell-T cell interaction partner [29]. Indeed, CTLA4 was recently shown to inhibit glycolysis in CD4+ Tcells, thus supporting our observations [30].

In a recent study, it was shown that the upregulation of BCL-6 represses genes encoding molecules involved in aerobic glycolysis that are upregulated during the effector phase of the immune response [29]. A connection between the expression of CTLA4 and BCL-6 has been discussed in the generation of follicular helper T cells [31], but if it is a common feature in modulating T cell metabolism remains unclear and has to be addressed in further experiments.

Since it is known that the PI3K/AKT/mTOR pathway is required for metabolism, we analyzed the contribution of AKT and mTOR, both involved in signaling processes known to regulate cellular metabolism [6, 32, 33]. Inhibition of mTOR with rapamycin showed no effect on lactate production, while the inhibition of AKT completely abrogated lactate production. Additionaly, we show for the first time that activation of AKT upregulates the expression of LDH, the enzyme which converts pyruvate to lactate. This observation was confirmed by specific functionally different AKT inhibitors which all abrogated the expression of LDH.

Moreover, we observed an increase in GLUT1 expression and glucose uptake upon tetramer stimulation which was also described before in primary human T cells [5, 6]. In contrast to these previous studies, the upregulation of GLUT1 and glucose uptake in OT1 CD8+ T cells was not AKT-dependent. These contradictory results were also described in a recent study by Macintyre and coworkers [8], showing that inhibition of AKT had no effect on glucose uptake after stimulation of P14 TCR tg T cells with gp33 peptide. Thus, we confirm these previous results showing that AKT has no impact on the uptake of glucose [8]. However we additionally show that AKT is required for the upregulation of lactate dehydrogenase expression. Therefore, we suggest that GLUT1 upregulation and glucose uptake are regulated in an AKT-independent manner whereas lactate production strongly requires the activation of AKT. It was shown that phosphoinositol-dependent protein kinase 1 (PDK1), an upstream activator of AKT, is responsible for upregulation of glucose uptake independent of the PI3K/AKT pathway [8]. Since the role of PDK1 was assessed in T cell blasts in the presence of high IL-2 concentrations, there is a strong temporal separation from our system. We clearly observe the upregulation of LDH in parallel with the activation of AKT within 48 h upon stimulation. This leads to the hypothesis that AKT activation upon stimulation induces upregulation of LDH whereas IL-2 production induces an AKT independent upregulation of glucose uptake via PDK1.

Surprisingly the inhibition of mTOR by rapamycin had no effect on lactate production, since it was described that mTOR is one of the crucial players in nutrient sensing in mammalian cells. Previous studies revealed an important role of mTOR in the regulation of differentiation into CD4+ T cell subsets like Th1, Th2 and Th17 cells [34, 35]. Furthermore we observed no activation of HIF1α (data not shown) which was proposed to be a link between mTOR and upregulation of glycolytic enzymes [36]. Therefore we could conclude that mTOR activation is not required for upregulation of glycolysis in CD8+ T cells.

## Conclusion

In summary, we show that antibody stimulation did not induce a significant increase in glycolytic metabolites and hence no shift towards glycolysis. The observed drop in ATP levels might be the cause and the consequence of the resulting apoptosis. Another possibility is that apoptotic processes which where induced by antibody stimulation, inhibit the metabolic reprogramming of T cells.

Tetramer stimulation of CD8^+^ T cells leads to the induction of aerobic glycolysis in order to fulfill the increased energy demands following T cell activation, which is IL-2 dependent and counteracted by the upregulation of CTLA4 . We show that AKT plays a major role in aerobic glycolysis via upregulation of LDH. Further experiments are required to identify other possible targets of AKT and to identify other players regulating T cell metabolism. The study by MacIntyre and coworkers [8] suggests that PDK1 is the master regulator of T cell metabolism by phosphorylating members of the AGC kinase family, like RSK, PKCs, and SGKs independent of the PI3K/AKT pathway. Recently a new molecule was identified called lymphocyte expansion molecule (LEM, [32]), which was shown to promote antigen specific CD8+ T cell expansion, effector function, and memory cell generation. LEM was observed to regulate the protein complexes of the oxidative phosphorylation pathway in the inner membrane of the mitochondria thereby upregulating the generation of reactive oxygen species which play a crucial role in T cell proliferation. Nevertheless the whole mechanism how this modulation effects T cell behaviour remains unclear.

The activation of the metabolic regulator AMPK appears to be not required for the shift of metabolism towards enhanced glycolysis since it is switched off shortly after stimulation. A recent study suggested that AMPK may be involved in the generation of memory CD8^+^ T cells. It was shown that addition of the drug metformin, a strong activator of AMPK, leads to reduced proliferation and an enhanced development of memory CD8^+^ T cells [37]. In contrast to CD4+ T cells mTOR appears to be dispensable for upregulation of glycolysis and induction of proliferation in CD8+ T cells. Therefore inhibition of AKT or an upregulation of CTLA4 could be possible targets for preventing the overreaction and exhaustion of T cells as seen in chronic viral infections, like HIV or HCV. Until now some metabolic inhibitors have been shown to suppress T cell responses in EAE, asthma, and graft versus host disease [38–40]. This leads to the conclusion that changes in T cell metabolism can alter T cell expansion and differentiation making metabolic regulation a powerful target for treatment of a large variety of diseases like immune-related diseases characterized by hyperactive T cells.

## Methods

### Mice, cell purification, stimulation

OT-I TCR transgenic (tg) mice were maintained in pathogen free conditions. All experiments were performed with samples taken from euthanized animals in accordance with the German National Guidelines for the Use of Experimental Animals (Animal Protection Act, Tierschutzgesetz, TierSchG). Animals were handled in accordance with the European Communities Council Directive 86/609/EEC. All possible efforts were made to minimize animal suffering and the number of animals used. Splenic CD8^+^ T cells from OT-I TCR tg mice were purified and stimulated as previously described [2]. CTLA4-/-/OT-I mice were kindly provided by M. Brunner-Weinzierl. Crosslinking of CTLA-4 (CD152) on C57BL/6 CD8+ T cells was performed using latex microspheres coated with antibodies. In brief, 107 microspheres/ml were suspended in PBS with CD3 (0.75 μg/ml), CD28 (2.5 μg/ml), CTLA-4 or a hamster isotype control antibody (A19-3, 8 μg/ml) and incubated for 1 h at 37 °C, followed by washing in PBS and blocking with complete media. CD8+ T cells (1.5x10^6^/ml) were stimulated at a ratio of 1:1 with antibody-coupled microspheres in the presence of IL-12 (5 ng/μl). Specificity of crosslinking of CD152 with antibody-coupled microspheres was controlled by stimulating naive CD8+ T cells (CD8+ CD62Lhigh) of OT-1 CTLA4−/− mice with CD3, CD28, and CTLA-4 or CD3, CD28, and isotype control-coupled microspheres.

### Human T cells

Approval for these studies was obtained from the Ethics Committee of the Medical Faculty at the Otto-von-Guericke University, Magdeburg, Germany. Informed consent was obtained in accordance with the Declaration of Helsinki. Peripheral blood mononuclear cells were isolated as previously described [40]. The Jurkat E6.1 T cell line was maintained in RPMI 1640 medium [BioChrom] supplemented with 10 % heat inactivated Fetal Bovine Serum (FBS) [PAN] and cultured at 37 °C and 5 % CO_2_ in humidified atmosphere.

### Metabolic analysis

CD8^+^ T cells were cultured in RPMI 1640 medium containing 10 % FCS (PAN Biotech), 100 U/ml penicillin, 100 μg/ml streptomycin (all from Biochrom AG), and 50 μM 2-ME in 48-well plates at a concentration of 4 x 10^6^ cells/well. Cells were left unstimulated or stimulated with either CD3/CD8 mAbs or OT-I tetramers at 37 °C. Cells were harvested after 2 h and 4 h. The cells were immediately resuspended in 600 μl ice cold Methanol/Chloroform (2:1). The subsequent metabolic extraction, measurements, and analysis were performed as previously described [41].

### Lactate and IL2 production

Stimulated OT-I T cells were harvested at indicated timepoints and analyzed for lactate production with Lactate assay kit (Biocat) measuring colorimetric changes at 450 nm according manufacturers instruction and the production of IL2 with ELISA MAX™ Standard Kit (Biolegend) using a microplate reader.

### Glucose uptake

OT-I T cells were stimulated for 24 h with OT-1 tetramers and then analyzed with glucose uptake cell-based assay kit (Cayman Chemical Company). Cells were harvested and starved for 15 min in PBS at 37C°. Afterwards fluorescent labelled glucose (150 μg/ml, 2-NBDG) was added to the cells for 15 min. After subsequent washing, the cells were additionally stained with pacific blue - Annexin V (Biolegend) and PI (Biolegend) and analyzed via FACS.

### Inhibitors and Western blotting

CD8^+^ T cells were either left untreated or stimulated with CD3/CD8 mAbs or OT-I tetramers. The inhibitors AKT V (1 μM) AKT-VIII (2 μM) AKT XII (5 μM;all Calbiochem) or Rapamycin (2 mM) were directly added to the samples after stimulation. Anti-AMPKα, anti-phospho(p)AMPKα (Thr172), Anti-p-ERK1/2, anti-pS6K (all Cell Signaling) and anti-β-actin (clone AC-15) (Sigma-Aldrich) were used for Western blotting.

### Flow cytometry

CD8^+^ T cells were stimulated with agonistic antibody coated microspheres for the indicated time, washed once and stained with CD25-AF647 [Biolegend] for 15 min at 4 °C. After one washing, samples were analyzed on FACSCantoII using FACSDiva software [BD].

### Statistics

All experimental results were analyzed for statistical relevance with ANOVA, *p* < 0.05 was considered significant.

## Abbreviations

AKT/PKB, Proteinkinase B; AMPK, AMP kinase; ATP, adenosine triphosphate; CTLA4, Cytotoxic T-lymphocyte-associated Protein 4; GLUT1, Glucose transporter 1; IL-2, Interleukin 2; LDH, Lactate dehydrogenase; LKB1, Liverkinase B1; mTOR, Mammalian target of rapamycin; TCA cycle, Tricarboxylic acid cycle; TCR, T cell receptor

## Additional file

Additional file 1: Figure S1. Purified CD8+ T cells were treated with OT-I-streptamers for the indicated time periods. Samples were analyzed by Western blotting using the indicated Abs to determine the inhibition of mTOR (A) and AKT (B). Purified CD8+ T cells were treated with OT-I-streptamers for 24 h in presence or absence of aIL-2. Samples were analyzed for pSTAT5 activation to determine the function of aIL-2 antibody (C) Toxicity was assessed for the inhibitors rotenone and oxamate and the aIL-2 antibody by AnnexinV PI staining 24 h after stimulation (D). (TIF 189 kb)
