# Supplementary figures and images for: Early changes in the metabolic profile of activated CD8+ T cells
Source: BMC Cell Biol. 2016 Jul 7;17:28. doi: 10.1186/s12860-016-0104-x (PMC4937576; doi:10.1186/s12860-016-0104-x)

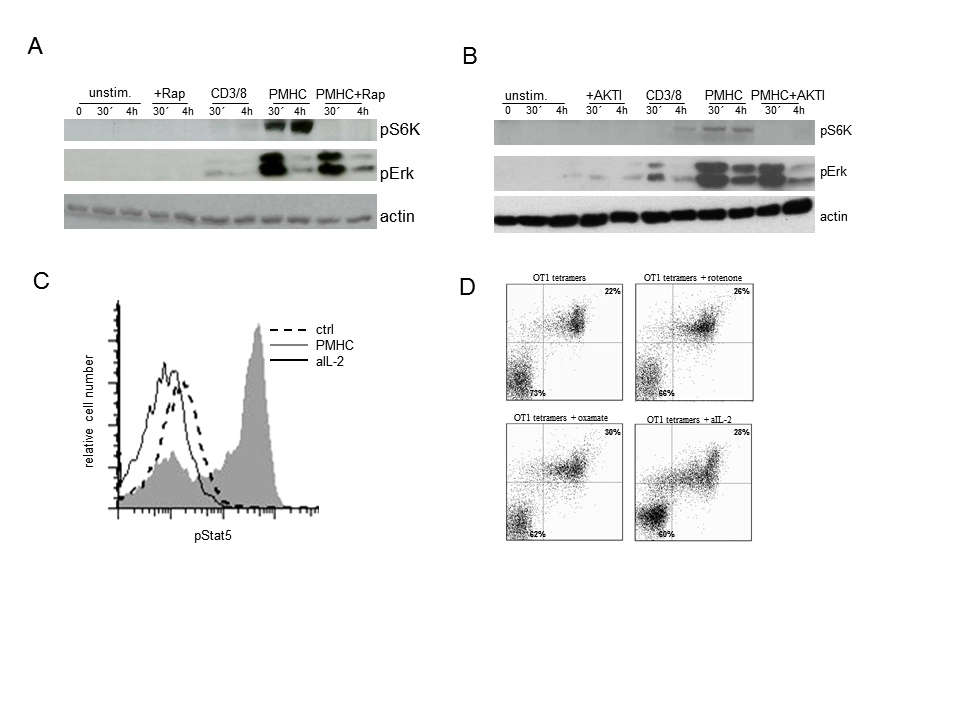

Supplement: Additional file 1: Figure S1. — Purified CD8+ T cells were treated with OT-I-streptamers for the indicated time periods. Samples were analyzed by Western blotting using the indicated Abs to determine the inhibition of mTOR (A) and AKT (B). Purified CD8+ T cells were treated with OT-I-streptamers for 24 h in presence or absence of aIL-2. Samples were analyzed for pSTAT5 activation to determine the function of aIL-2 antibody (C) Toxicity was assessed for the inhibitors rotenone and oxamate and the aIL-2 antibody by AnnexinV PI staining 24 h after stimulation (D). (TIF 189 kb) [file 12860_2016_104_MOESM1_ESM.tif]
